# Study protocol for a randomized controlled trial of the NEIVATECH virtual reality system to improve visual function in children with anisometropic amblyopia

**DOI:** 10.1186/s12886-022-02466-z

**Published:** 2022-06-07

**Authors:** L. Leal Vega, D. P. Piñero, C. J. Hernández Rodríguez, A. Molina Martín, L. Morales-Quezada, A. I. Vallelado Álvarez, J. F. Arenillas Lara, M. B. Coco Martín

**Affiliations:** 1 Group of Applied Clinical Neurosciences and Advanced Data Analysis, Department of Medicine, Dermatology and Toxicology, University of Valladolid, Av. Ramón y Cajal, 7, 47005 Valladolid, Spain; 2 Group of Optics and Visual Perception, Department of Optics, Pharmacology and Anatomy, University of Alicante, Alicante, Spain; 3 Clinical Optometry Unit, Department of Ophthalmology, Vithas Medimar International Hospital, Alicante, Spain; 4 Department of Physical Medicine and Rehabilitation, Spaulding Rehabilitation Hospital, Harvard Medical School, Boston, USA; 5 Pediatric Ophthalmology Unit, Department of Ophthalmology, University Clinical Hospital of Valladolid, Valladolid, Spain; 6 Stroke Unit and Stroke Program, Department of Neurology, University Clinical Hospital of Valladolid, Valladolid, Spain

**Keywords:** Anisometropic amblyopia, Neural plasticity, Gamification, Dichoptic training, Perceptual learning, Virtual reality

## Abstract

**Background:**

Interest in developing alternative methods for the treatment of amblyopia has long been a topic of interest among clinicians and researchers, as prescribed occlusion and penalization therapies do not always provide an effective response and are associated with a high risk of recurrence and non-compliance. Here, we present the protocol of a small-scale RCT to evaluate the safety and clinical efficacy of a novel VR-based system designed to provide binocular vision training to children with anisometropic amblyopia.

**Methods:**

We aim to recruit a total of 60 children with anisometropic amblyopia aged 5-17 years with no previous treatment for amblyopia other than refractive correction from the pediatric ophthalmology units of the University Clinical Hospital of Valladolid and the Vithas Medimar International Hospital of Alicante. Children who meet the eligibility criteria and consent to participate will be randomly assigned to a three-month intervention group of 18 half-hour in-office therapy sessions with the NEIVATECH system (group A) or to a parallel group receiving 2 hours of conventional patching per day at home for the same period of time (group B). Assessments of visual function will be carried out before the intervention and at 1, 2 and 3 months, with changes in distance BCVA being the primary outcome measure to be considered. Patient safety, compliance, satisfaction and acceptance to treatment will also be assessed after therapy as other valuable outcome measures. In addition, a rsfMRI scan will be performed on a subgroup of 5 patients from each group at the pre-intervention visit and at the post-intervention visit to test the effects of both therapies on neural plasticity in the visual cortex.

**Discussion:**

The NEIVATECH system has been conceived as a serious game designed to provide binocular vision training to anisometropic amblyopic children by complementing the concepts of perceptual learning, dichoptic training and gamification in an immersive VR environment. We hope that this novel approach may lead to greater improvements in vision performance than those provided so far by conventional patching in anisometropic amblyopic children.

**Trial registration:**

This protocol was registered with ClinicalTrials.gov (NCT04819386) on 29 March 2021.

**Supplementary Information:**

The online version contains supplementary material available at 10.1186/s12886-022-02466-z.

## Background

Amblyopia has traditionally been defined as a reduction in the best-corrected visual acuity (BCVA) of one or both eyes in the absence of obvious organic disease [1]. This reduction in BCVA is known to be due to an abnormal binocular experience originating during the critical period of visual system development, which may be caused by a misalignment of the eyes (strabismic amblyopia), a significant difference in the refractive error of the eyes (anisometropic amblyopia) or the presence of an ocular condition that prevents adequate visual stimulation (deprivation amblyopia) [2].

A wide range of large cohort and population-based studies have concluded that the global prevalence of amblyopia averages between 3 and 5%, making it the world’s leading cause of visual impairment in children and young adults [3, 4].

Current treatment of amblyopia emphasizes patching or penalizing the non-amblyopic eye to force the use of the affected eye, and although this is an effective method of improving visual acuity (VA), it has been associated with residual monocular and binocular deficits and a high risk of recurrence and non-compliance [5–7]. Furthermore, it has been argued that this monocular approach rarely results in the establishment of binocular function and is not considered valid in amblyopic patients who are beyond the critical period of visual system development [8, 9]. All these reasons have led to a substantial increase in research in the last decades on new treatment approaches that can satisfy both children and adults, with perceptual learning, video games and binocular treatment methods being the most relevant trends aimed at improving binocular fusion and reducing interocular suppression commonly present in amblyopia [10].

### Perceptual learning

Perceptual learning can be defined as an evolution in the discernment of a stimulus array after repetitive exposure or practice with this array [11].

According to Hebbian theory, robust and synchronous activation in neuronal populations can lead to enhanced synaptic strength between the neurons comprising those populations [12], so that repeated presentation of visual stimuli may induce such neuroplastic changes in sensorimotor networks to promote, in this case, visual functions affected by amblyopia.

In this regard, a number of visual tasks have been explored as a means to apply perceptual learning, including letter identification in noise, position discrimination in noise, Gabor filter-based edge detection, contrast detection, etc. [13].

In their comprehensive literature review of perceptual learning applied to amblyopia, Levi and Li reported on the relative efficacy of various of these visual tasks on both trained task performance and VA as measured by the Snellen chart, with five of the twelve studies reviewed showing improvements in post-test outcomes, of which four employed contrast detection practice and one examined extended positional acuity learning in children [14]. Similarly, Polat et al. found a 2-fold improvement in contrast sensitivity function (CSF) and letter recognition tasks when a series of 77 adults with amblyopia participated in a perceptual learning trial with Gabor patches, suggesting that perceptual learning promotes modifications of early neural processes localized beyond the site of convergence of both eyes and that residual plasticity is present in the adult visual system [15].

Overall, perceptual learning has been shown to significantly improve visual functions in amblyopia across a wide range of tasks, including: VA, CSF [14, 15], Vernier acuity [16, 17], positional acuity [18] and first and second order letter identification [19, 20].

It is worth mentioning that, although some studies on perceptual learning in the treatment of amblyopia have been strongly criticized (mainly due to the small sample size used, the lack of long-term follow-up, and the need, in most cases, for perceptual learning tasks to be performed in a clinical laboratory setting), other studies that have analysed the effects of perceptual learning on neural plasticity using functional magnetic resonance imaging (fMRI) techniques have shown that it can modify the functional specializations of visual cortical areas [21–23].

### Binocular methods

Since the loss of binocularity is one of the defining features of amblyopia, the importance of binocular approaches to its therapy have recently received widespread attention.

One of the best-known treatment methods for amblyopia that directly addresses binocular dysfunction by promoting binocular vision and reducing inhibitory interactions within the visual cortex is dichoptic training, which consists of prolonged periods of viewing stimuli under conditions of increased contrast for the amblyopic eye [24, 25].

In this context, Li et al. demonstrated that about 9 hours of dichoptic film viewing on a passive 3D display spread over 6 sessions during a two-week time period resulted in an improvement of 1 to 4 lines of VA in amblyopic children aged 4 to 10 years [26], while conventional occlusion therapy, in comparison, require about 120 hours to achieve 1 line of VA improvement in amblyopic children who have already been treated with spectacles over a period of 12 to 16 weeks [27].

Another study evaluating the efficacy of binocular training in improving VA in children and adults with amblyopia was a non-controlled, non-randomized clinical trial (RCT) in which kinemograms of random dots were presented dichoptically to both eyes, with the visual task being to identify the direction of target motion. After completing an average of 14.5 training sessions over a period of 4 to 6 weeks, the patients’ mean VA improved by 0.34 logarithm of the minimum angle of resolution (LogMAR) and was maintained 6 months after cessation of binocular training [28].

### Video games

In recent years, modern technology advancements in the field of informatics have enabled researchers to use appropriate interactive software to improve visual performance. To this end, visual stimuli can be presented to the user in two ways: on a computer screen or in a fully immersive environment generated by specific technological equipment.

Recent research has shown that playing video games using the amblyopic eye for 40-80 hours produces improvements in VA, positional acuity, spatial attention and stereopsis, while performing other monocular activities does not cause this effect [29]. This may be due to the attentional or motivational effects associated with video game playing [30]. In particular, the recovery in VA observed in this study was at least 5-fold faster than would be expected with the same hours of occlusion treatment.

It has also been observed that dichoptic video games can induce greater improvements in VA than when played monocularly. For example, in a crossover study in which 18 adults with amblyopia were allocated to train with an engaging video game presented on head-mounted video goggles for 1 h per day over 2 weeks with the fellow eye patched (group A) or play the same game dichoptically (group B), dichoptic training led to greater improvements in VA than monocular training. In addition, when patients in group A were crossed over to play dichoptically there was a more pronounced improvement in VA [31]. These results are consistent in amblyopic children, in which there has been reported that 20 hours of game play, either monocularly, with the fellow eye patched, or dichoptically, with reduced contrast for the fellow eye, are sufficient to improve VA on average by 0.14 logMAR (≈ 38%) for the dichoptic training group (*n* = 10) and by 0.06 logMAR (≈ 15%) for the monocular training group (*n* = 11), respectively [32].

Other studies, such as the binocular treatment of amblyopia using videogames (BRAVO) RCT, in which 115 children, teenagers and adults with amblyopia were assigned to play a dichoptic contrast block-falling video game on an iPod Touch for 1 hour a day during a 6-week period or a placebo video game found no significant improvement in VA between the two groups [33]. Similarly, a study by the Pediatric Eye Disease Investigator Group (PEDIG) showed that the improvement in VA obtained with 16 weeks of binocular video games on an iPad device for 1 hour per day was not as good as with 2 hours of prescribed occlusion therapy in 385 amblyopic children aged 5 to 12 years and 13 to 16 years with no history of previous treatment for amblyopia [34, 35].

As this is a controversial topic, there is therefore a scientific need, as well as a clear market niche, to find new binocular games that provide a clear benefit compared to conventional patching in children with amblyopia.

## Methods

### Hypothesis and aims

We hypothesise that 9 hours of therapy with the NEIVATECH system, which combines dichoptic training with perceptual learning tasks and gamified elements in an immersive virtual reality (VR) environment, will provide greater improvements in visual function in children with anisometropic amblyopia than conventional patching.

To test our hypothesis, we intend to conduct a small-scale RCT in which 60 anisometropic amblyopic children aged 5-17 years with no history of treatment for amblyopia other than refractive correction alone will be randomly assigned to receive 18 half-hour active vision therapy sessions with the NEIVATECH system over a period of 3 months (group A) or 2 hours of daily patching at home for the same period of time (group B).

The primary outcome measure to be considered will be changes in distance BCVA before and after therapy. Secondary outcome measures will include near BCVA, CSF, binocular vision, stereopsis, fusional vergence, near convergence point and accommodative facility. Other valuable outcome measures to consider after the intervention will be satisfaction, adherence, acceptance and safety of the therapy. In addition, resting-state fMRI (rsfMRI) will also be performed on a subgroup of 5 patients from each group at the pre- and post-intervention visit to provide a rational explanation for the visual improvements obtained with the NEIVATECH system, if any.

### Study design

This is a multicentre, open-label, small-scale RCT involving two tertiary hospital centres with an on-site pediatric ophthalmology unit in their facilities. The protocol for this study was designed according to the SPIRIT (Standard Protocol Elements: Recommendations for Interventional Trials) 2013 Statement (Supplementary file 1). Patients will be offered to participate in the study once they have been found to meet all inclusion criteria without fulfilling any exclusion criteria (Table 1).

**Table 1:** Eligibility criteria for participation in the NEIVATECH RCT

Inclusion criteria	
- Children aged between 5 and 17 years. - Willingness to perform the assigned therapy and to carry out the established visits. - No history of previous treatment for amblyopia other than optimal refractive correction for at least 2 months prior to inclusion in the study. - BCVA in the amblyopic eye of ≤0.1 logMAR with at least 1 logMAR line difference between the two eyes of the same patient. - Presence of anisometropia defined as a difference in spherical equivalent between the two eyes of ≥1.5 dioptres.	
Exclusion criteria	
- Active eye disease. - Previous ocular surgery. - BCVA in the amblyopic eye of ≥0.70 logMAR. - Presence of strabismic or deprivation amblyopia. - Presence of microtropia or intermittent strabismus. - Presence of irregular cornea due to astigmatism or ectatic corneal disease. - Presence of psychiatric or neurological disorders or cognitive impairment.	

Prior to participation, trained research staff will obtain written informed consent from the patient or the patient’s parents or legal guardians depending on whether the patient is older or younger than 16 years of age. For the rsfMRI study, patients will have to meet specific eligibility criteria and sign a separate informed consent form to undergo testing (Table 2).

**Table 2:** Eligibility criteria for participation in the rsfMRI study

Inclusion criteria	
- Inclusion in the NEIVATECH RCT. - Written informed consent for rsfMRI.	
Exclusion criteria	
- Claustrophobia. - Pregnancy or breastfeeding. - Presence of a pacemaker or other cardiac device. - Presence of an external or implantable insulin pump. - Presence of metallic implants or intracorporeal foreign bodies.	

Patients may leave the study once they have completed all visits as outlined in the protocol or whenever they wish by contacting the principal investigator and signing a waiver form.

To minimize the risk of bias, this study will be blinded to the endpoint evaluators through the adoption of a PROBE (Prospective Randomized Open, Blinded End-point) design.

### Study setting

The setting in which the patients will be recruited and the study visits will be carried out will be the pediatric ophthalmology units of the University Clinical Hospital of Valladolid and the Vithas Medimar International Hospital of Alicante.

On the other hand, the setting in which the necessary equipment will be installed to carry out the active visual therapy sessions with the NEIVATECH system will be a consultation room of the Department of Medicine of the University of Valladolid and the Department of Optics, Pharmacology and Anatomy of the University of Alicante.

All rsfMRI scans that are intended to be performed as part of the study will take place in the Philips Achieva 3.0 T X-Series Scanner of the Laboratory of Instrumental Techniques (LTI) of the University of Valladolid.

### Sampling

Consecutive sampling of all patients attending the pediatric ophthalmology units of the hospital centres planned for recruitment who met the eligibility criteria will be carried out over a period of 12 months (May 2022 to April 2022). The contact details of all patients who consent to participate will be registered in order that they can be contacted to arrange in-office active vision therapy sessions and scheduled visits. Participants will be assigned to the experimental or control group by block randomization using a computer-generated list of random numbers in order to ensure equal treatment allocation. Once randomized, each patient recruited in Valladolid will also be offered the possibility of participating in the rsfMRI study until the proposed number in each group is reached.

Sample size estimation for this study was carried out using the GRANMO online platform (https://www.imim.es/ofertadeserveis/software-public/granmo/), establishing a minimum distance BCVA difference to be detected in both groups before and after the intervention of 0.15 logMAR, a follow-up loss of 10% and a common standard deviation (SD) of 0.195 logMAR. According to this, a sample size of 30 patients per group would be required, considering an alpha error of 0.05 and a statistical power of 80%. This result is consistent with the sample size used in previous studies in this area [36–38].

### Intervention

**Fig. 1:**
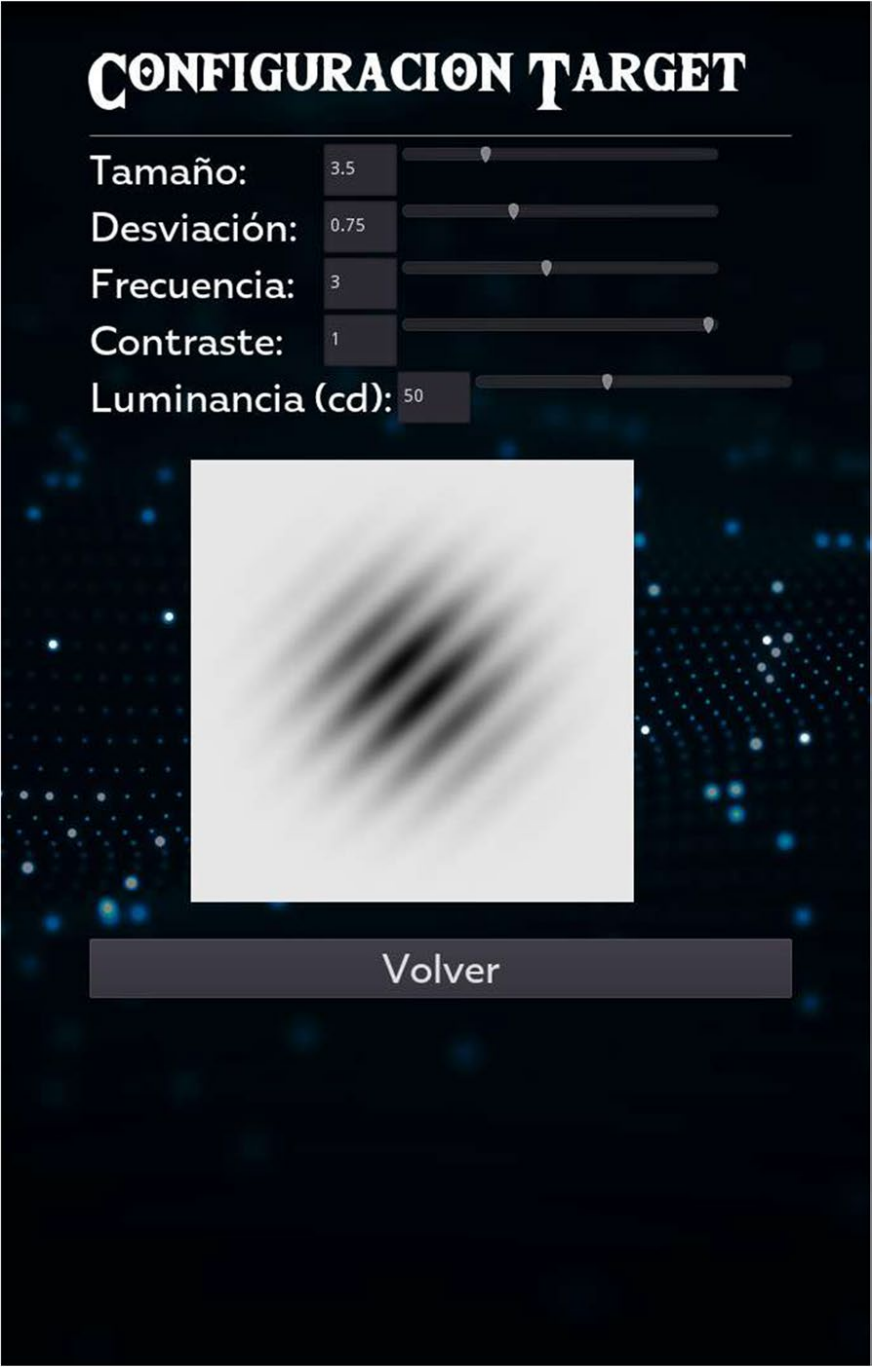
Menu to configure the characteristics of the visual stimuli to be presented to the user in the VR environment

The NEIVATECH VR-based system has been conceived as a Serious Game designed to provide dichoptic training to amblyopic children by discriminating the direction of Gabor patches presented with different contrast to each eye as a perceptual learning task. Patients have to identify the same Gabor patch as the one presented at the start of the game, all in different VR environments where immersion will be achieved using the HTC Vive Pro Head Mounted Display (HMD) and interaction will be achieved using the VR Developer Mount system. The NEIVATECH system is also equipped with software to configure the characteristics of the visual stimuli presented to users according to their vision (Fig. 1) and to record the results obtained by the user in each session, facilitating the analysis and evaluation of individual progress over time.

### Patient and public involvement

**Fig. 2:**
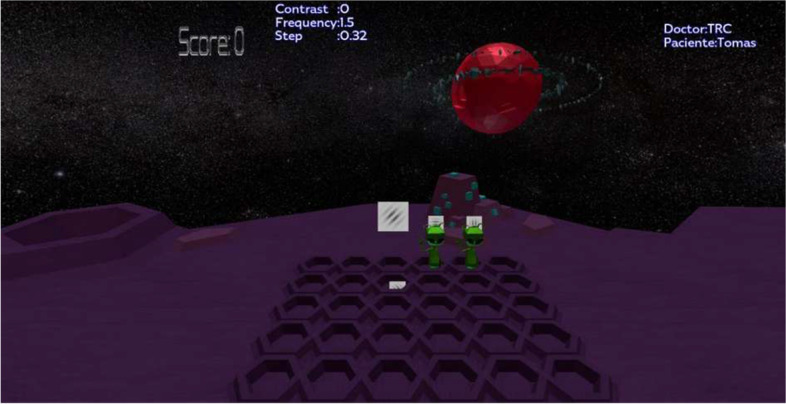
Example of a VR scenario incorporated into the NEIVATECH system

A total of 22 children (14 boys, 8 girls, mean age: 10.36 ± 1.76 years) participated in the design of the VR scenarios that make up the Serious Game by providing feedback on the initially designed scenarios and suggestions on the incorporation of new elements to make them more attractive to the target population. For this purpose, a visual analogue scale created by the research team was used to record patients’ opinions and requests. Figure 2 shows an example of a scenario (Space) with the discrimination task to be performed.

### Study overview

Participation in this RCT will essentially involve five visits (screening, pre-intervention visit, one-month visit, two-month visit and post-intervention visit), in addition to the visits that patients assigned to group A will have to make to carry out the active vision therapy sessions with the NEIVATECH system. These sessions will be 18 in total, will have an average duration of half an hour and will be distributed as follows: five sessions in the first week (Monday to Friday), three sessions in the second week (Monday, Wednesday and Friday) and a maintenance session in the following weeks (on Wednesdays). This is because it has been shown that most of the improvement obtained with visual training in amblyopia occurs during the first weeks of treatment [39–41]. Thus, patients assigned to group A will have to perform 10 sessions of active vision therapy with the NEIVATECH system during the first month of treatment, 4 during the second month and 4 during the third month. On the other hand, patients assigned to group B will have to comply with 2 hours of daily patching during the 3 months of the study.

**Fig. 3:**
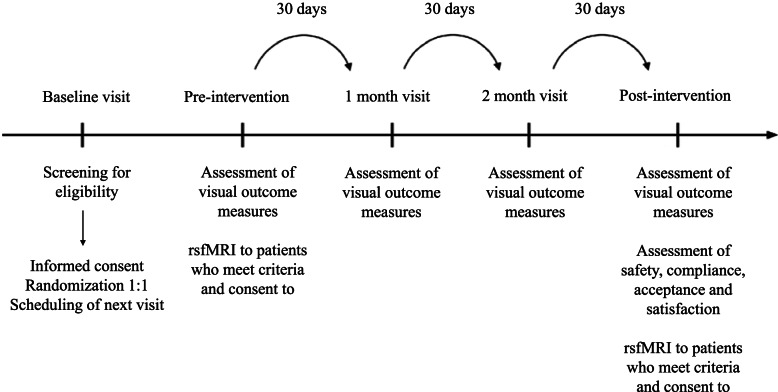
Diagram of the NEIVATECH RCT protocol

In addition, a subgroup of 5 patients from group A and 5 patients from group B will have to make two additional visits to the LTI of the University of Valladolid before and after the intervention for a rsfMRI scan (Fig. 3).

### Pre-intervention (day 0)

Once the patient has been found to meet the eligibility criteria required for participation and the informed consent form has been signed, he/she will be seen in consultation prior to receiving the assigned intervention for a comprehensive eye examination including cycloplegic autorefraction and assessment of near and distance BCVA (monocular and binocular, logMAR scale, using the ETDRS chart), CSF (monocular, using the CSV-1000 test for spatial frequencies of 3, 6, 12 and 18 cycles per degree), binocular vision (using the Worth’s four-dot test), stereopsis (using the TNO stereoscopic test), fusional vergence (break and recovery), accommodative facility (measured as the number of changes made to achieve optimal focusing using a spherical flap of ±2 dioptres when the patient looks at a near optotype: VA 0.2 logMAR) and near convergence point (measured as the closest distance in centimetres in reference to the patient’s nasal root at which he/she is able to maintain single vision).

### Mid-intervention (day 30 and 60)

After 1 and 2 months of therapy, the patient will be referred for re-evaluation of the visual outcome measures.

### Post-intervention (day 90)

After 3 months of treatment, the patient will be referred for reassessment of the same visual outcome measures, in addition to measures of compliance, satisfaction, acceptance and adverse reactions to therapy. Patient compliance with the NEIVATECH system will be determined by the number of sessions attended by each patient during the 3 months of the study, while compliance with the patch will be assessed by means of an adherence record to be completed by parents or legal guardians. Patient satisfaction and acceptance to the new therapy will be assessed using the User Satisfaction Evaluation Questionnaire (USEQ) [41] and the Technology Acceptance Model (TAM) questionnaire [42]. Finally, adverse reactions to therapy will be assessed using the standard and pediatric versions of the Simulator Sickness Questionnaire (SSQ) [43, 44], depending on whether the patients are older or younger than 12 years of age.

### Follow-up

Six months after the post-intervention visit, patients will be seen at the recruiting centres’ pediatric ophthalmology units according to daily clinical practice for reassessment of the visual outcome measures considered in the study.

### Data analyses plan

A professional biostatistician has been consulted to design the data analysis plan for the study, in which descriptive analysis of all data will be provided, including means and SDs or medians for continuous variables and proportions for categorical variables.

Variables will be analyzed to assess distribution/normality (Kurtosis and Skewness) and by histogram analysis to establish whether they will be analyzed with parametric or non-parametric tests. On the one hand, parametric data will be analyzed using the unpaired T-test, Analysis of Variance (ANOVA) for comparison between groups and multiple linear regression to predict values according to the variables of interest. On the other hand, non-parametric data will be analyzed using the Mann-Whitney U test or the Chi-squared test, the Kruskal-Wallis H test and other non-parametric methods.

For the purposes of the rsfMRI study, analyses of grey matter, white matter diffusion and functional connectivity will be performed. To quantify the variation between the metrics obtained in the two acquisitions (pre-intervention and post-intervention), a coefficient of variation of (Mt_0_ − Mt_1_ / Mt_0_) × 100 will be used, where M will be the metric considered, t_0_ will correspond to the first acquisition (pre-intervention visit) and t_1_ will correspond to the second acquisition (post-intervention visit).

### Dissemination plan

As a dissemination plan, results obtained from the study will be presented at national and international conferences and through peer-reviewed publications. Furthermore, two PhD students will publish and defend dissertations related to the results of the study.

## Discussion

This small-scale RCT adds to the short list of studies that have evaluated VR technologies for the binocular treatment of amblyopia [36–38, 45–47]. As it primarily targets children, different Game-based elements and dynamics have been incorporated into the system to increase patient compliance with treatment. An attempt will also be made to correlate the presumed improvements in visual function to be obtained with rsfMRI findings at the pre-intervention visit and at the post-intervention visit. It should be noted that this study has important limitations, such as the need for the child and family to travel to the space where the NEIVATECH VR-based system will be installed for the active vision therapy sessions in the experimental group, the absence of a “real” control group under a sham intervention for ethical reasons and the impossibility to objectively confirm compliance to patching in the control group in the absence of electronic occlusion dose monitors (ODMs).

## Supplementary Information

**Additional file 1.**

## Data Availability

The information obtained from the NEIVATECH sessions, patch adherence records and clinical tests performed will only be shared with the research team for analysis purposes, respecting the privacy of the participants in accordance with the Organic Law 3/2018, of 5 December, on Protection of Personal Data and Guarantee of Digital Rights. These data will be encrypted and stored in a secure electronic database that can only be accessed by the research team with a specific username and password. Data sets analysed during the study will only be available to study investigators, endpoint evaluators and members of the Research Ethics Committees of the participating centres, although the corresponding author may make them available to other investigators upon reasonable request (e-mail: juanfarenillas@gmail.com).

## Abbreviations

ANOVA: Analysis of variance
BCVA: Best-corrected visual acuity
BRAVO: Binocular treatment of amblyopia using videogames
CSF: Contrast sensitivity function
fMRI: Functional magnetic resonance imaging
HMD: Head mounted display
LogMAR: Logarithm of the minimum angle of resolution
LTI: Laboratory of instrumental techniques
ODMs: Occlusion dose monitors
PEDIG: Pediatric eye disease investigator group
PROBE: Prospective randomized open, blinded end-point
RCT: Randomized controlled trial
rsfMRI: Resting-state functional magnetic resonance imaging
SD: Standard deviation
SPIRIT: Standard protocol items: recommendations for interventional trials
SSQ: Simulator sickness questionnaire
TAM: Technology acceptance model
USEQ: User satisfaction evaluation questionnaire
VA: Visual acuity
VR: Virtual reality
